# The Impact of Musical Engagement on Mental Health: A Comparison Between Amateur Musicians, Professional Musicians, and Non-musicians

**DOI:** 10.1192/j.eurpsy.2026.10746

**Published:** 2026-07-31

**Authors:** I. Ekmekci, M. H. Aksu, B. Coşar

**Affiliations:** Gazi University, Ankara, Türkiye

## Abstract

**Introduction:**

The concept of “musicians’ health” has emerged as a specific field of study (Bonde et al. 2018). Amateur engagement with music or music listening has been repeatedly associated with beneficial effects on both physical and mental health (Theorell & Kreutz 2012).

**Objectives:**

This study aimed to compare depression, anxiety, stress, somatization, and somatic symptom amplification levels among professional musicians, amateur musicians, and individuals with no involvement in music, in order to clarify whether different types of musical engagement influence mental health outcomes.

**Methods:**

This descriptive study included three groups: (1) professional musicians whose occupation and livelihood depend on music, (2) amateur musicians engaged in music as a hobby, and (3) individuals who had never practiced music either professionally or as a hobby. Participants were recruited via online survey links. After informed consent, they completed a sociodemographic form, the Depression Anxiety Stress Scale-21 (DASS-21), the Somatosensory Amplification Scale( SSAS), and the Patient Health Questionnaire-15 (PHQ-15). Data were analyzed using one-way ANOVA and Tukey post-hoc tests.

**Results:**

Sociodemographic characteristics are summarized in Table 1. As shown in Table 2, there were significant differences among groups in DASS-21 depression, anxiety, stress, SSAS, and PHQ-15 scores. Professional musicians had the highest mean scores across all measures.

**Table 1.** Sociodemographic characteristics of participants Table 1. Long description.

Variables	Amateur musicians (n=236)	Professional musicians (n=100)	Non-musicians (n=88)
Gender (F/M)	122 (52%) / 114 (48%)	32 (32%) / 68 (68%)	49 (56%) / 39 (44%)
Smoking	59 (25%)	31 (31%)	17 (19%)
Alcohol use	77 (32%)	36 (36%)	38 (57%)
Substance use	1 (0%)	2 (2%)	1 (1%)
Age (mean±SD)	47.57±14.35	42.06±15.26	46.51±12.68
Education (yrs)	16.84±3.53	16.45±3.79	18.15±3.02

**Table 2.** Comparison of psychological measures between groups Table 2. Long description.

Scale / Subscale	Amateur musicians	Professional musicians	Non-musicians	F	p	Post-hoc (Bonferroni)
PHQ-15 (somatization)	7.00±4.90	9.15±5.78	6.94±4.72	6.88	0.001	1–2 (p=0.001); 2–3 (p=0.009)
SSAS (Somatosensory Amplification)	23.43±6.68	26.03±7.03	24.01±6.85	5.78	0.003	1–2 (p=0.016); 2–3 (p=0.004)
DASS-21 Depression	4.82±3.77	6.34±4.47	4.88±3.80	5.50	0.004	1–2 (p=0.004)
DASS-21 Anxiety	2.97±2.64	4.79±3.64	3.22±2.89	13.49	<.001	1–2 (p<0.001); 2–3 (p=0.001)
DASS-21 Stress	5.50±3.41	6.86±4.10	5.48±3.50	5.40	0.005	1–2 (p=0.005)

**Conclusions:**

Professional musicians demonstrated significantly higher levels of depression, anxiety, stress, somatization, and somatosensory amplification compared with both amateur musicians and non-musicians. Elevated somatization in this group is noteworthy and may be linked to comorbid stress, anxiety, and depression. Professional musicians may therefore represent a population at risk for psychosomatic conditions, highlighting the need for targeted preventive and therapeutic approaches.

**Disclosure of Interest:**

None Declared

## Long descriptions

### Table 1. Long description

The table consists of four columns: Variables, Amateur musicians (n=236), Professional musicians (n=100), and Non-musicians (n=88).

* Gender (F/M): Amateur musicians 122 (52%) / 114 (48%); Professional musicians 32 (32%) / 68 (68%); Non-musicians 49 (56%) / 39 (44%).

* Smoking: Amateur musicians 59 (25%); Professional musicians 31 (31%); Non-musicians 17 (19%).

* Alcohol use: Amateur musicians 77 (32%); Professional musicians 36 (36%); Non-musicians 38 (57%).

* Substance use: Amateur musicians 1 (0%); Professional musicians 2 (2%); Non-musicians 1 (1%).

* Age (mean plus or minus S D): Amateur musicians 47.57 plus or minus 14.35; Professional musicians 42.06 plus or minus 15.26; Non-musicians 46.51 plus or minus 12.68.

* Education (yrs): Amateur musicians 16.84 plus or minus 3.53; Professional musicians 16.45 plus or minus 3.79; Non-musicians 18.15 plus or minus 3.02.

Navigate back to Table 1..

### Table 2. Long description

The table consists of seven columns: Scale / Subscale, Amateur musicians, Professional musicians, Non-musicians, F, p, and Post-hoc (Bonferroni).

* P H Q-15 (somatization): Amateur musicians 7.00 plus or minus 4.90; Professional musicians 9.15 plus or minus 5.78; Non-musicians 6.94 plus or minus 4.72. F equals 6.88, p equals 0.001. Post-hoc shows significant differences between groups 1-2 (p=0.001) and 2-3 (p=0.009).

* S S A S (Somatosensory Amplification): Amateur musicians 23.43 plus or minus 6.68; Professional musicians 26.03 plus or minus 7.03; Non-musicians 24.01 plus or minus 6.85. F equals 5.78, p equals 0.003. Post-hoc shows significant differences between groups 1-2 (p=0.016) and 2-3 (p=0.004).

* D A S S-21 Depression: Amateur musicians 4.82 plus or minus 3.77; Professional musicians 6.34 plus or minus 4.47; Non-musicians 4.88 plus or minus 3.80. F equals 5.50, p equals 0.004. Post-hoc shows significant difference between groups 1-2 (p=0.004).

* D A S S-21 Anxiety: Amateur musicians 2.97 plus or minus 2.64; Professional musicians 4.79 plus or minus 3.64; Non-musicians 3.22 plus or minus 2.89. F equals 13.49, p is less than 0.001. Post-hoc shows significant differences between groups 1-2 (p is less than 0.001) and 2-3 (p=0.001).

* D A S S-21 Stress: Amateur musicians 5.50 plus or minus 3.41; Professional musicians 6.86 plus or minus 4.10; Non-musicians 5.48 plus or minus 3.50. F equals 5.40, p equals 0.005. Post-hoc shows significant difference between groups 1-2 (p=0.005).

Navigate back to Table 2..

